# Sugarcane bagasse pretreatment using three imidazolium-based ionic liquids; mass balances and enzyme kinetics

**DOI:** 10.1186/1754-6834-5-62

**Published:** 2012-08-24

**Authors:** Sergios Kimon Karatzos, Leslie Alan Edye, William Orlando Sinclair Doherty

**Affiliations:** 1 School of Chemistry, Queensland University of Technology, GPO Box 2434, Brisbane, QLD, 4001, Australia; 2 Sugar Research and Innovation, Queensland University of Technology, GPO Box 2434, Brisbane, QLD, 4001, Australia

**Keywords:** Ionic liquids, Pretreatment, Sugarcane bagasse, Enzyme hydrolysis

## Abstract

**Background:**

Effective pretreatment is key to achieving high enzymatic saccharification efficiency in processing lignocellulosic biomass to fermentable sugars, biofuels and value-added products. Ionic liquids (ILs), still relatively new class of solvents, are attractive for biomass pretreatment because some demonstrate the rare ability to dissolve all components of lignocellulosic biomass including highly ordered (crystalline) cellulose. In the present study, three ILs, 1-butyl-3-methylimidazolium chloride ([C4mim]Cl), 1-ethyl-3-methylimidazolium chloride ([C2mim]Cl), 1-ethyl-3-methylimidazolium acetate ([C2mim]OAc) are used to dissolve/pretreat and fractionate sugarcane bagasse. In these IL-based pretreatments the biomass is completely or partially dissolved in ILs at temperatures greater than 130°C and then precipitated by the addition of an antisolvent to the IL biomass mixture. For the first time mass balances of IL-based pretreatments are reported. Such mass balances, along with kinetics data, can be used in process modelling and design.

**Results:**

Lignin removals of 10% mass of lignin in bagasse with [C4mim]Cl, 50% mass with [C2mim]Cl and 60% mass with [C2mim]OAc, are achieved by limiting the amount of water added as antisolvent to 0.5 water:IL mass ratio thus minimising lignin precipitation. Enzyme saccharification (24 h, 15FPU) yields (% cellulose mass in starting bagasse) from the recovered solids rank as: [C2mim]OAc(83%) > >[C2mim]Cl(53%) = [C4mim]Cl(53%). Composition of [C2mim]OAc-treated solids such as low lignin, low acetyl group content and preservation of arabinosyl groups are characteristic of aqueous alkali pretreatments while those of chloride IL-treated solids resemble aqueous acid pretreatments. All ILs are fully recovered after use (100% mass as determined by ion chromatography).

**Conclusions:**

In all three ILs regulated addition of water as an antisolvent effected a polysaccharide enriched precipitate since some of the lignin remained dissolved in the aqueous IL solution. Of the three IL studied [C2mim]OAc gave the best saccharification yield, material recovery and delignification. The effects of [C2mim]OAc pretreatment resemble those of aqueous alkali pretreatments while those of [C2mim]Cl and [C4mim]Cl resemble aqueous acid pretreatments. The use of imidazolium IL solvents with shorter alkyl chains results in accelerated dissolution, pretreatment and degradation.

## Background

Lignocellulosics, whether in the form of dedicated energy crops such as switchgrass, agricultural residues such as sugarcane bagasse, or from forestry residues, present an abundant renewable energy resource. With world energy demand predicted to increase in the near future [1], and fossil fuel reserves being depleted and non-renewable, biomass resources have drawn much attention as renewable and sustainable feedstocks for alternative fuels and chemicals.

The conversion of the lignocellulosic biomass polysaccharides to ethanol fuel and/or other products of fermentation (e.g. butanol) involves hydrolysis, fermentation and product separation. However, these polysaccharide molecules are not readily hydrolysed since they are contained in the chemically recalcitrant and structurally complex lignocellulosic matrix. This matrix is designed by nature to protect the plant organism from chemical and biological attack. Therefore, a pretreatment step is added to the process prior to hydrolysis in order to improve saccharification of the polysaccharides.

Most pretreatment technologies are either physical (e.g., size comminution, steam explosion and hydro-thermolysis) or chemical, utilizing organic solvents, acids or alkalis [2-4]. These chemical pretreatment technologies occur by either acid or alkali mechanisms at high temperatures and pressures. Acid pretreatments are known to dissolve hemicellulose and cleave arabinosyl glycosidic linkages whereas alkali pretreatments dissolve lignin and cleave acetyl bonds [5].

Recently, ionic liquids (ILs) have drawn a great deal of attention as solvents for pretreatment of lignocellulosics. ILs are a class of organic salts that are liquid at temperatures below 100°C. Many ILs are non-volatile, non-explosive, stable at a wide range of temperatures and reaction-condition severities and compatible with a wide array of organic and inorganic functional chemicals and solvents [6]. Pretreatment of the lignocellulosic matrix with ionic liquids occurs by the dissolution-then-precipitation of solids which exhibit reduced crystallinity and improved enzyme saccharification of cellulose [7-11]. For a thorough review which focuses on dissolution of biomass, lists 74 ILs that have been tested for the ability to dissolve cellulose, wood, pulp, hemicellulose and lignin and also includes other solvents of relevance to woody biomass processing, *viz.*, choline urea mixture - a deep eutectic salt, and N-methylmorpholine-N-oxide monohydrate - a conventional cellulose solvent, the reader is referred to Pinkert et al. [12]. While it is expected that ionic liquids impart compositional changes and cleave covalent bonds of the original biomass in order to dissolve its components, little is known about the nature of these changes and how they vary with varying ionic liquids. In addition there are few accounts of full mass closures of ionic liquid pretreatments. For example Arora et al. [13] reported mass balances of [C2mim]OAc treated switchgrass (160°C, 3 h, 3% loading) and Sun et al. [14] reported mass balances for [C2mim]OAc treatment of southern yellow pine (110°C for 16 h, 5% loading). These few accounts do not provide direct comparisons between ILs while they do not account for IL masses and do not differentiate between hemicellulose components in the recovered solids. Three most cited ionic liquids for biomass pretreatment are the imidazoliums: 1-butyl-3-methylimidazolium chloride or [C4mim]Cl, 1-ethyl-3-methylimidazolium chloride or [C2mim]Cl and 1-ethyl-3-methylimidazolium acetate or [C2mim]OAc. This study a) investigates the structural and compositional characteristics of sugarcane bagasse pretreated with each of the aforementioned ionic liquids and b) provides full mass balances for these pretreatment processes.

## Results and discussion

### Dissolution of bagasse in ILs

The dissolution of bagasse in the three ILs under identical conditions (150°C, 90 min and 5% mass bagasse in IL, conditions as in previous work [10]) was investigated and the results are shown in Table 1. It has to be noted that the [C2mim]OAc dissolution at this loading (5% mass) was very viscous and hard to stir.

**Table 1:** Effect of ionic liquid choice on bagasse dissolution

**IL**	**mg**	**% starting mass**				**Mass fraction**
	**Starting mass**	**Undissolved solids**	**Dissolved-and-recovered solids**	**Dissolved unrecovered**	**Total dissolved**	**Unrecovered/ total dissolved**
[C4mim]Cl	224	37	43	21	63	0.33
[C2mim]Cl	224	15	26	60	85	0.70
[C2mim]OAc	224	4	56	40	96	0.42

Masses expressed on dry basis.

The effect of cation size is exhibited when comparing [C4mim]Cl with [C2mim]Cl. In agreement with the literature [12], the smaller [C2mim] cation imparts higher dissolution (85% *cf* 63%) and this may be due to the enhanced penetration of the smaller solvent molecule resulting in higher dissolution. The effect of anion is studied by comparing [C2mim]Cl to [C2mim]OAc. The acetate anion seems to favour dissolution as opposed to losses. Moreover, losses may be exacerbated due to the fact that dissolution (96%) is well into the last recalcitrant bagasse fraction. It is probable that by reducing the severity of the treatment conditions, the ratio of dissolution to losses will be improved. Acetate has a higher hydrogen bond basicity than chloride [15] and thus its ability to disrupt hydrogen bonds and dissolve cellulose is higher. This positive correlation between the hydrogen bond basicity (β, a Kamler-Taft solvation parameter) of the IL anion and the IL’s ability to dissolve cellulose or lignocellulose is reported in the literature [15]. The superior dissolving capacity of [C2mim]OAc has also been reported by Sun et al. [14] who measured 93.5% dissolution extent of southern yellow pine in [C2mim]OAc and only 26% in [C4mim]Cl under the same conditions (particle size 0.25 – 0.50 mm, 5% mass loading, 110°C for 16 h).

### Fractional precipitation by stepwise addition of water

The potential of using incremental amounts of water to fractionally precipitate IL-dissolved bagasse was tested on three ILs and the mass balances of each process determined. The fractional precipitation process was designed to yield a polysaccharide-rich and a lignin-rich fraction using two incremental additions of water (and acidification to pH ≤1.0 and a third addition of water to precipitate remaining dissolved material).

The first water addition used for partial precipitation of dissolved solids resulted to a water : IL mass ratio of 0.5. Preliminary qualitative studies based on visual observations of fractional precipitation with water (where bagasse soda lignin -prepared in house- and Avicel were used) indicated that this water amount should precipitate all cellulose and keep lignin in solution for water-[C4mim]Cl mixtures and possibly also for water-[C2mim]OAc mixtures. It was also indicated that lignin precipitation was complete at a water:[C4mim]Cl mass ratio of 2.0. However native bagasse lignin dissolved in IL is likely to have different properties to lignin extracted with aqueous NaOH and then dissolved in IL. Maximum lignin recovery was ensured by acidification to a pH ≤1.0 and the addition of a further 1.5 IL mass equivalents of water. Water-[C2mim]Cl solutions are assumed to behave in a similar manner to water-[C4mim]Cl solutions.

Bagasse pretreatments with [C4mim]Cl, [C2mim]Cl and [C2mim]OAc under identical conditions (35 min at 150°C, 5% bagasse in IL (2.5% for [C2mim]OAc)) imparted partial dissolution and the polysaccharide rich solid fractions (SF1) were recovered using water addition (water : IL mass ratio of 0.5). These conditions are deliberately less severe than in the first dissolution experiments, so as to lessen the degradation reactions and allow for the recovery of sufficient SF1 solid mass for mass balance and further analysis. The SF1 solids were washed and freeze-dried prior to analysis and enzyme saccharification. Bagasse was extracted with water and ethanol prior to treatment since better mass balance closures are obtained by removing non-structural molecules which can interfere with characterisation of solid and liquid fractions.

### Compositional analysis

The composition of SF1 fractions from each IL pretreatment (at 150°C for 35 min with 25 min temperature ramp) are shown in Table 2. These solids contain both the undissolved bagasse and the dissolved-then-precipitated bagasse and the proportion of each has not been measured. However they have been measured (at 150°C for 90 min, see Table 1). Of course the extents of biomass dissolution and degradation to material not recovered in the solid fraction (“losses” to liquid fraction) would both be expected to increase with temperature and treatment time.

**Table 2:** Compositional analysis of SF1 solids from pretreatment of ethanol-extracted bagasse with three different ILs

**Sample**	**% dry mass**									**Ratios**	
	**Mass recovery**	**Ash**	**AIL**	**ASL**	**Total lignin**	**Glucan**	**Xylan**	**Arabinan**	**Acetyl**	**Arabinan /Xylan**	**Acetyl /Xylan**
		**±0.4**	**±0.4**	**±0.1**		**±0.2**	**±0.5**	**±0.08**	**±0.05**		
**Untreated (extracted)**	100	3.1	20.8	5.4	26.2	44.9	22.2	1.50	3.11	0.07	0.14
**[C4mim]Cl**	90	3.5	20.8	5.4	26.1	47.6	20.5	1.06	2.96	0.05	0.14
**[C2mim]Cl**	48	6.1	25.0	3.8	28.7	53.4	11.0	0.60	1.75	0.05	0.16
**[C2mim]OAc**	66	5.7	9.9	6.0	15.8	68.2	13.3	1.58	1.42	0.12	0.11

AIL: Acid insoluble lignin, ASL: Acid Soluble Lignin.

In the mass balance experiments reported in Table 2, the extent of dissolution has been estimated indirectly from the solids “losses” (and ratios of dissolved mass to losses derived from Table 1). For the [C4mim]Cl dissolution a ratio of unrecovered to total dissolved mass of 1:3 was observed (Table 1). This ratio was found to be 1:2.3 for extracted bagasse. The “losses” incurred for [C4mim]Cl are ca. 10% mass (Table 2), thus the dissolution extent in these experiments for [C4mim]Cl is estimated to be around 23% mass. The other two ILs are known to effect different ratios of dissolution extents to losses (Table 1), and the losses incurred in these ILs (34% - 52%, Table 2) suggest that the dissolutions are close to complete. These estimates were confirmed visually; it was observed that [C4mim]Cl contained a very large amount of undissolved fibre, [C2mim]OAc only a small amount and [C2mim]Cl almost none.

For [C4mim]Cl-treated solids, the compositional changes are small with a slight cellulose enrichment. This is primarily because only ca. 23% of the bagasse dissolved. For [C2mim]Cl the SF1 is rich in cellulose and lignin and low in hemicellulose saccharides. Given the fact that [C2mim]Cl dissolves and degrades bagasse components faster than [C4mim]Cl, it is not surprising that the [C2mim]Cl solids are low in hemicellulose components. The [C2mim]OAc SF1 is rich in cellulose and arabinose and it is low in lignin, xylan and acetyl content. The [C2mim]Cl SF1 is also rich in cellulose but also rich in lignin. Hemicellulose appears to have been substantially removed by [C2mim]Cl treatment.

It is tempting to speculate that the acetate IL and chloride ILs cleave different bonds. It appears that the acetate IL effects preferential delignification and deacetylation whereas the chloride ILs effect preferential removal of arabinose and xylan. In terms of solids composition the acetate IL appears to effect a similar outcome to aqueous alkali treatment while the chloride ILs produce a similar outcome to dilute acid treatments. This is a key finding. Note that the aqueous acid and alkali treatments involve biomass dissolution and decomposition while IL treatments involve dissolution decomposition and precipitation. Thus, the chemical processes involved are different, but the gross compositional changes are similar.

The acetate may be expected to be basic and be more reactive than chloride because acetate is a stronger base than chloride (acetic acid pKa = 4.75 *cf.* HCl pKa = −7.0) but these chemical behaviours do not necessarily hold true for non-aqueous IL solutions.

### Mass recovery of bagasse components after pretreatment

Mass balances for bagasse components after the first water addition (water:IL mass ratio of 0.5, yielding SF1 and LF1) of the three IL pretreatments ([C2mim]OAc, [C4mim]Cl, [C2mim]Cl) were determined and are shown in Table 3.

**Table 3:** Mass balance of bulk biomass and of biomass components from three treatments with different ILs

**Dry mass (mg)**	**Bulk biomass**	**Ash**	**Lignin (AIL + ASL)**	**Glucan**	**Xylan**	**Arabinan**	**Acetyl**	**HMF**	**Furfural**
[C4mim]Cl									
Untreated	**1461**	46	383	656	324	22	46	n/a	n/a
SF1	1318	47	344	627	270	14	39	n/a	n/a
LF1	92	n/d	0.4*	16	60	10	7	0.6	1.2
LF1 oligo				13	58	2	6		
Total mass recovered	**1411**	47	344	636	317	24	46		
[C2mim]Cl									
Untreated	**1346**	42	353	605	299	20	42	n/a	n/a
SF1	643	39	185	343	71	4	11	n/a	n/a
LF1	446	n/d	3*	188	214	14	28	1.2	1.4
LF1 oligo				175	204	3	27		
Total mass recovered	**1089**	39	188	516	267	20	39		
[C2mim]OAc									
Untreated	**699**	22	183	314	155	10	22	n/a	n/a
SF1	463	26	73	316	62	7	7	n/a	n/a
LF1	88	n/d	17*	1	85	5	n/d	1.1	1.8
LF1 oligo				bdl	83	5	n/d		
Total mass recovered	**551**	26	90	316	125	12	7		

Masses (average of duplicates).

Masses expressed on dry basis. Glucan is considered equal to cellulose, HMF (Hydroxymethyl furfural) is expressed as glucan equivalents and furfural as xylan equivalents, SF1: solid fraction 1, recovered from the first water addition (water:IL mass ratio = 0.5), LF1: liquid fraction 1, recovered from the first water addition (water:IL mass ratio = 0.5), LF1 oligo: oligosaccharides in LF1, n/d: not determined, n/a: not applicable, bdl: barely detectable.

The estimates of standard deviation (absolute, based on duplicate IL pretreatments, 3 df) of the recovery (and analysis) of these components (as%mass starting component) in SF1 are 2% for glucan, 2% for xylan, 3% for arabinan, 1% for acetyl and 2% for lignin (acid soluble + acid insoluble). In LF1, these estimates of standard deviation for the oligosaccharides are 1% for glucan, 2% for xylan, 18% for arabinan and 3% for acetyl and for the monosaccharides they are 0.2% for glucan, 0.2% for xylan, 15% for arabinan and 0.7% for acetyl. The unacceptably high standard deviation for arabinan is attributed to its very low concentrations in the liquid fractions. *This mass does not represent all the lignin mass in the LF1 but only the recoverable lignin mass in the sum of SF2 and SF3 precipitates.

The composition of the SF1 solids and the LF1 liquids (sum of monosaccharides and oligosaccharides) were determined. The total mass accounted for by the mass balance determination protocol differs greatly between ILs. For [C4mim]Cl the original bagasse mass accounted for is 97% mass (1411 mg out of 1461 mg) and this may be attributed to the low dissolution extent achieved by this treatment. For [C2mim]Cl, the mass recovery is 81% mass (1089 mg out of 1346 mg) and for [C2mim]OAc 79% mass (551 mg out of 699 mg). For all 3 IL treatments, the remaining bagasse mass (not accounted for by mass balance determinations) is mainly (more than half) lignin that was not recoverable from the liquid fraction followed by polysaccharides (glucan followed by xylan for the chloride ILs and xylan only for [C2mim]OAc). Note that in the liquid fraction of the [C2mim]OAc pretreatment, the biomass-derived acetyl content (representing ca. 3% of starting bagasse mass) is not detectable since the IL counter ion is acetate.

The percent mass of each starting bagasse component in each pretreatment fraction (*viz.* solid fraction, liquid fraction oligosaccharides and liquid fraction monosaccharides) is listed in Table 3. Note that the degradation products HMF and furfural represent a small fraction of the mass in the liquid fraction and are included in the mass balances listed in Table 3. as glucan and xylan equivalents.

For [C4mim]Cl, 95% mass of the starting cellulose, 83% of xylan and 90% of lignin are recovered in the solid fraction. Out of the 10% lignin in the liquid fraction, only 0.3% was recoverable (see Table 4), indicating that the vast majority of the lignin mass remaining in the liquid fraction after addition of 0.5 IL mass equivalents of water is in a form that cannot be recovered by further additions of water or acidification (i.e. it is soluble and likely low molecular weight material). Hemicellulose components are depolymerised preferentially while a big part of the arabinose (36%) is removed, all of which is in the monomeric form. The arabinofuranosyl glycosidic linkages are acid labile and consequently arabinose loss is a characteristic of acid treatments [5]. The fact that arabinose is found in the monomeric form indicates either that the arabinosyl groups removed are terminal or if they are not, that the ester bonding of arabinose to lignin is concomitantly cleaved.

**Table 4:** Mass recovery and lignin content of solids recovered from the liquid fraction after treatment with three ILs

	**mg**	**% mass**	**mg (% starting mass of lignin)**	
**Sample**	**Recovered mass**	**Lignin content**	**Lignin recovery**	**pH**
**[C4mim]Cl SF2**	1.2	31	0.4 (0.3)	6.1
**[C4mim]Cl SF3**	0.3	n/d	n/d	1.0
**[C4mim]Cl TOTAL**	1.5		0.4 (**0.3**)	
**[C2mim]Cl SF2**	10.8	30	3.2 (1.5)	3.6
**[C2mim]Cl SF3**	0.3	n/d	n/d	0.3
**[C2mim]Cl TOTAL**	11.1		3.2 (**1.5**)	
**[C2mim]OAc SF2**	58.2	23	13.4 (9.9)	7.0
**[C2mim]OAc SF3**	12.2	26	3.2 (1.7)	1.0
**[C2mim])OAc TOTAL**	70.4		16.6 (**11.7**)	

Masses expressed on dry basis.

Mass balance analysis for [C2mim]Cl treatment reveals the same trends as for [C4mim]Cl but since dissolution is near complete in this IL the trends are more pronounced. Lignin and cellulose are the predominant components of the recovered solids reflecting the relative thermal and chemical stability of these polymers in solution when compared to hemicelluloses. Out of 78% mass arabinan in the liquid fraction, 55% mass is in monomeric form. Out of 50% lignin expected in the liquid fraction only 1.5% is recoverable.

Preferential hemicellulose removal resulting in lignin and cellulose enrichment of the treated solids is also a characteristic of dilute acid treatments. The chloride imidazolium ILs appear to remove arabinose preferentially. Note that in previous work [10], where the undissolved fraction of bagasse after [C4mim]Cl dissolution was analysed, it was shown that cellulose dissolved to a greater extent than hemicelluloses (the effect of dissolution only). The analysis of SF1 here indicates that hemicellulose is preferentially removed (the net effect of dissolution and reprecipitation). Cellulose is preferentially dissolved but at a high *DP* (degree of polymerisation) and mostly recovered by precipitation with the addition of water, whereas the little hemicellulose that dissolves, depolymerises and becomes soluble in the water-IL mixture (i.e. does not reprecipitate).

The mass distributions of bagasse components after [C2mim]OAc pretreatment are also shown in Table 3. While 100% mass of the original cellulose is recovered in the solid fraction the lignin content is reduced to 40% mass. Cellulose appears to get solubilised in a high *DP* form since it is all precipitated with the addition of water and none of it is found in the aqueous liquid fraction. These features indicate that [C2mim]OAc affords excellent cellulose preservation and substantial delignification. However, out of the 60% mass lignin extracted in the liquid fraction, only 11.7% was recoverable. Since arabinose content is comparatively high in the solid fraction and found in the oligomeric liquid fraction only, it can be concluded that arabinose is preserved and that no terminal arabinosyl groups have been removed (i.e. the arabinosyl glycosidic linkages in hemicellulose are stable in [C2mim]OAc). Finally the acetate content of the solid fraction is reduced by 70% mass indicating substantial deacetylation.

The arabinan recovery is inflated (115% mass) and this is attributed to the large standard deviation of arabinose measurements in the liquid fraction as discussed earlier.

Overall the [C2mim]OAc pretreatment affords distinctly different compositional changes to the chloride IL pretreatments. These distinct differences are delignification, deacetylation preservation of cellulose glycosidic bonds (as deduced from the absence of cellulose mass in the liquid fraction) and preservation of arabinosyl groups in hemicellulose. These differences are similar to the differences between acid and alkali aqueous pretreatments. Note that previous work by Fu et al. [16], where triticale straw was treated with six different ILs, is in agreement with the present study in that [C2mim]OAc affords extensive delignification while preserving the cellulose fraction of the treated biomass.

### ATR-FTIR

**Figure 1:**
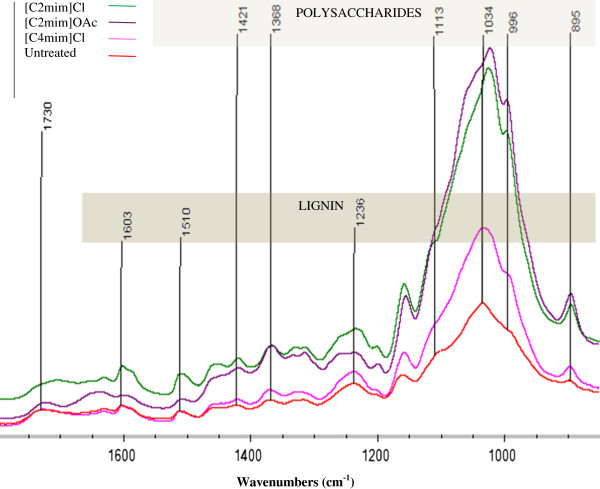
**FTIR spectra of bagasse treated with different ILs. **(absorbance – common scale).

The SF1 from each IL was also analysed using ATR-FTIR and the spectra are shown in Figure 1. Table 5 lists the assignments of the absorption bands of interest. In general, these spectra reflect the compositional characteristics discussed above. For example it is clearly discernible that the band absorbances which are characteristic of lignin are relatively low in the spectrum of SF1 treated with [C2mim]OAc when compared to other spectra.

**Table 5:** Assignments of FTIR-ATR absorption bands for bagasse

**Band position (cm**^**-1**^**)**	**Assignment**
**1730**	C = O stretching vibration in acetyl groups of hemicelluloses
**1600**	C = C stretching vibration in aromatic ring of lignin
**1510**	C = C stretching vibration in aromatic ring of lignin
**1421**	CH_2_ scissoring at C(6) in cellulose
**1368**	Symmetric C–H bending in cellulose
**1236**	C-O stretching vibration in lignin, xylan and ester groups
**1100**	O-H association band in cellulose and hemicelluloses (associated with crystalline cellulose)
**1030**	C-O stretching vibration in cellulose and hemicelluloses
**974**	C-O stretching vibration in arabinosyl side chains in hemicellulose
**895**	Glucose ring stretch, C1-H deformation

[17-20].

Infrared spectra were also used to estimate crystallinity of each SF1. The absorbance at 1421 cm^-1^ is viewed as typical of crystalline regions of cellulose and the absorption band at 893 cm^-1^ typical of amorphous regions; the ratio of these two bands represents a crystallinity index (CrI) also known as lateral order index [17]. The crystallinity indices of the solids recovered from all three IL treatments (0.19 for [C2mim]OAc , 0.21 for [C4mim]Cl and 0.37 for [C2mim]Cl) were significantly lower that of the untreated bagasse solids (0.88). Decrystalisation of cellulose is a known and unique effect of IL pretreatments [10]. The estimate of the standard deviation (absolute) for this crystallinity index-measurement is 0.02 (based on duplicate IL pretreatments, 3 df). These indices combined with the observed shift of the 1034 cm^-1^ band to lower wavenumber, represent a significant loss of crystallinity of cellulose after treatment in all three ILs. Note that despite only ca. 23% dissolution of bagasse in [C4mim]Cl the SF1 CrI is significantly lower than starting bagasse. As shown in previous work by the authors, the cellulose in the IL-undissolved fraction can also undergo decrystallisation [10].

### Enzyme saccharification

**Figure 2:**
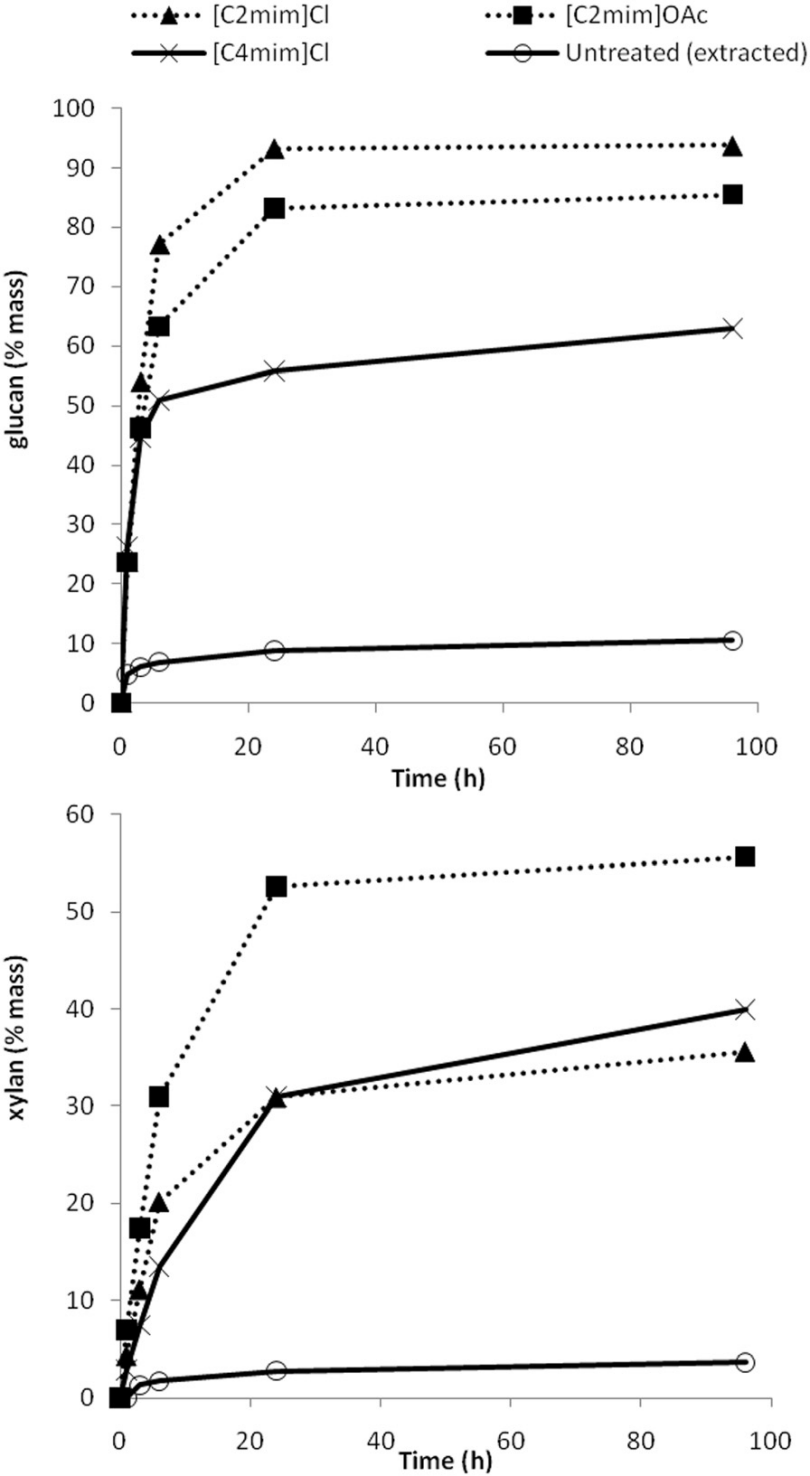
**Glucan and xylan saccharification of extracted bagasse treated with 3 ILs. **Glucan refers to glucan in pretreated solids and includes glucan equivalents of cellobiose released. Xylan refers to xylan in pretreated solids.

The progress of saccharification resulting from each IL treatment for cellulose and hemicellulose (as xylan) was monitored and is plotted in Figure 2. Initial rates of cellulose saccharification are very fast for all IL treatments, and this is the effect of cellulose decrystallisation which is common to all three treatments. The extent of saccharification is higher for the [C2mim]^+^ ILs than for the [C4mim]^+^ IL and of the [C2mim]^+^ salts, the chloride anion gives a higher saccharification yield. Given that [C2mim]OAc delignifies as well as decrystallises bagasse, it is surprising that [C2mim]Cl yielded a higher final saccharification. This is possibly due to the different lignin-hemicellulose bonds (as identified by compositional and infrared analysis and discussed above) that survive in the solids after these two pretreatments. The fast initial saccharification rates resulting from [C4mim]Cl treatment reflect the presence of structural changes while the low final saccharification yields are a consequence of a lower extent of dissolution and indicate the absence of compositional and covalent bonding perturbation in the undissolved solids.

Interestingly, the hemicellulose saccharification of [C2mim]OAc treated bagasse proceeds faster and closer to completion than for the other two ILs. This shows that the delignification achieved by [C2mim]OAc results in improved hemicellulose saccharification results, while for the chloride IL treated solids the hemicellulose is not as accessible to enzymes due to the persistence of lignin. It is likely that covalent linkages between lignin and hemicelluloses survive chloride IL treatment and limit the extent of saccharification of hemicelluloses.

**Figure 3:**
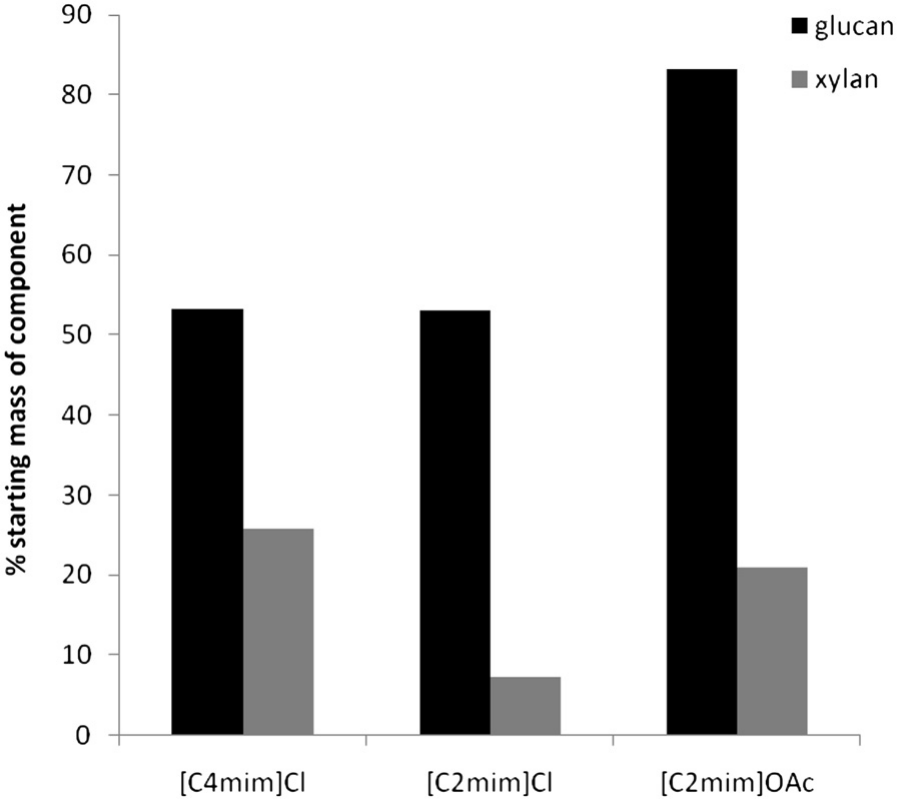
**Fraction of starting bagasse polysaccharides saccharified in 24 h (15 FPU g**^**-1 **^**glucan) after pretreatment in three ILs. **Glucan includes glucan equivalents of cellobiose released.

The cellulose and hemicellulose saccharification extents achieved by each IL at 24 h, as% mass theoretical in the starting bagasse (prior to pretreatment), are shown in Figure 3. Factoring in both the saccharification extent and the recovery of the starting polysaccharide mass in the pretreated solids, [C2mim]OAc affords the highest cellulose conversion due to a combination of rapid saccharification and no degradation of cellulose upon pretreatment. [C2mim]Cl and [C4mim]Cl have similar theoretical cellulose conversions. The much higher dissolution extent in [C2mim]Cl did not benefit the overall performance of this pretreatment since losses in [C2mim]Cl were much higher than in [C4mim]Cl. In terms of hemicellulose saccharification yield, [C4mim]Cl performs best mainly because more hemicellulose is preserved in polymeric and recoverable form.

In general and in agreement with the literature, for the cations, it appears that the shorter alkyl chain of [C2mim]Cl (*cf.* [C4mim]Cl) imparted faster dissolution and greater extent of saccharification. However higher dissolution rates were accompanied by higher degradation rates. The anion effect is greater since it imparts entirely distinct dissolution patterns. In the case of acetate compared to chloride, the acetate ion appears to impart a more alkali-resembling effect while the chloride ones a more acid-resembling effect.

### Lignin recovery

The second addition of water to the liquid fractions of the three IL pretreatments to a water:IL mass ratio of 2.0 yielded solid fractions (SF2). Acidification of the liquid fractions and a third water addition yielded some additional precipitate (SF3). For all SF2 and SF3 samples, lignin content was measured by the acetyl bromide method and FTIR spectra were obtained. SF2 and SF3 lignin contents and recovered masses are reported in Table 4. The weight of all liquid fraction precipitates and their lignin content was used to determine the total amount of lignin recoverable from the liquid fraction of each IL pretreatment. The lignin recovery from the liquid fraction of all three IL pretreatments was low (0.3% to 11.7% mass of starting lignin).

**Figure 4:**
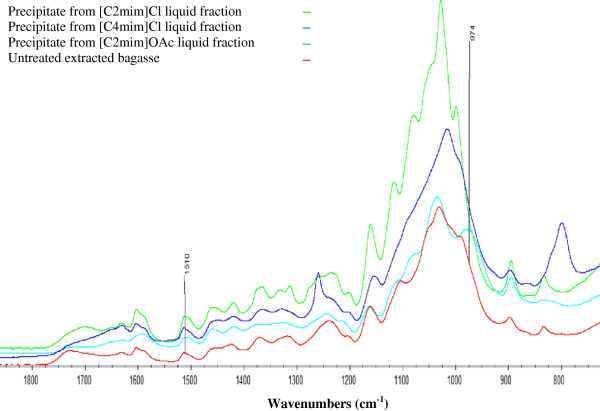
**FTIR spectra of precipitate recovered after precipitation in 3.5 water : IL mass ratio (acidified to pH ≤1) in three ILs. **(absorbance – common scale).

The ATR-FTIR spectra for the solids precipitated from each IL pretreatment from the 3.5 mass ratio water addition (SF3) are shown in Figure 4. Spectra of SF2 precipitates were also obtained but were not different to those of the SF 3 precipitates. These spectra have intense absorbances at the hemicellulose characteristic bands between 1175 cm^-1^ and 1000 cm^-1^. This indicates that the majority of the non-lignin component of these fractions (ca. 70% mass) is comprised of hemicellulose. This is in agreement with previous discussion demonstrating the preservation of lignin-hemicellulose bonding upon biomass dissolution in ILs. The [C2mim]OAc precipitate in particular has a strong characteristic band at 974 cm^-1^ indicative of arabinosyl groups. This again is in agreement with previous discussion supporting preservation of covalent bonds to arabinosyl moieties during [C2mim]OAc dissolution whilst they are labile in chloride IL dissolutions.

Undoubtedly, a large proportion of the dissolved lignin remains in the water-IL mixture even after addition of more water (3.5 water : IL mass ratio) and lowering of the pH to ≤1.0. In this fractional precipitation approach there are difficulties in quantitatively recovering lignin since only a small fraction of it precipitates. Furthermore the precipitate is far from pure lignin as it can contain up to 70% mass hemicellulose. While the cellulose fraction can be obtained in an enriched and extensively decrystallised form, the poor lignin recovery and co-precipitation of hemicelluloses argues against the use of this approach in an industrial setting.

### Ionic liquid recovery

The recovery of the ionic liquid solvent forms part of the mass balance closure and it was measured using ion chromatography of the liquid fraction of each pretreatment. The recovery of all ions appears to be full (100% mass) within experimental error. The standard deviation of repeat analyses was 2%. At least under laboratory conditions and scale little or no degradation of IL occurs and the IL could be completely recovered. Covalent bonding of acetate ions from [C2mim]OAc to biomass [21] and the reaction of imidazole cations with saccharides to form imidazole glycosides [22] may occur but at the low biomass concentrations used here the magnitudes of these reactions are small by comparison to the experimental error in the measurement of IL recovery.

## Conclusions

Mass balances are reported for pretreatment of bagasse with [C4mim]Cl, [C2mim]Cl and [C2mim]OAc. The acetate IL preferentially removes lignin and acetyl, while it preserves arabinosyl groups, resembling the effects of aqueous alkali pretreatments. Chloride ILs impart an opposite effect and resemble dilute acid pretreatments. The shorter IL alkyl chain accelerated the dissolution, pretreatment and degradation. Regulated addition of water-antisolvent affected a polysaccharide rich precipitate by maintaining dissolved lignin in all three ILs. In terms of saccharification yields, material recovery and delignification, [C2mim]OAc ranks as the most suitable IL for biomass pretreatment. The mass of all ILs used was fully recovered. Future work should focus on elucidating the mechanisms of lignocellulose dissolution and depolymerisation in acetate and chloride-based ILs. In addition the distinct chemistries of these IL solvents might be used to development an understanding of the reactions involved in other solvent based biomass pretreatments.

## Methods

### Bagasse

Sugarcane bagasse (Rocky Point sugar mill, Pimpama, Queensland) comprising long cuticle fibres and core pith particles (mostly ca. 5 mm to 50 mm) was air dried for a week on metal trays then size reduced (to <10 mm) with a knife mill before being mixed and subsampled by the cone and split method and stored at 4°C. Before use the bagasse was ground (to <2 mm) using an electric lab mill (Retsch SM100, Haan, Germany). The material was ground for ca. 1 min per batch to avoid excess heating, placed on top of two brass sieves (0.5 mm and 0.25 mm) and in a sieve shaker for 20 min, and the fraction collected between the two sieves was used as the starting material. Moisture content was measured gravimetrically (convection oven, 105°C, overnight) before every use and was 10 ± 1% mass except where otherwise indicated.

### Chemicals

The ionic liquids (1-butyl-3-methylimidazolium chloride [C4mim]Cl (≥ 95%) melting point as per MSDS (m.p.) 73°C, 1-ethyl-3-methylimidazolium chloride [C2mim]Cl (≥ 95%) m.p. 80°C, and 1-ethyl-3-methylimidazolium acetate [C2mim]OAc (≥ 90%) m.p. –20°C, Sigma-Aldrich, NSW) were all dried in a vacuum oven (at 80°C – 90°C, ca. 4 mm Hg, > 12 h) prior to use. Initial moisture content (at the time of weighing the IL for each use) was typically ca. 2% of total mass for [C4mmim]Cl and 1% for [C2mim]Cl and [C2mmim]OAc as measured by Karl Fischer titration. At this point it is worth noting that although the m.p. of neat [C4mim]Cl is 73°C (as per the MSDS provided by the manufacturer), its 2% moisture content was sufficient to maintain it in liquid phase at room temperature. Cellulose (Avicel PH-101), dimethyl sulphoxide (DMSO) (99.9%) and Karl Fischer HYDRANAL titrant 2E and solvent E were purchased from Sigma-Aldrich (Sydney, NSW). Cellulase / β-glucosidase mixture (Accelerase 1000) was purchased from Genencor (Danisco A/S, Denmark). Water was Millipore-filtered and deionised (Milli-Q-plus) to a specific resistivity of 18.2 μS at 25°C. All other solvents and chemicals were analytical grade.

### Bagasse soda lignin preparation

Bagasse soda lignin was prepared by soda pulping of bagasse (175°C, 2 h, bagasse 10% mass, NaOH 10% mass) and precipitating the resulting black liquor with acid (2 M H_2_SO_4_) add to reduce the pH to 3.0. The precipitate was then redissolved in aqueous NaOH (10% mass) and reprecipitated by addition of acid to reduce the pH to 3.0. The recovered lignin solids were washed and dried (40°C, vacuum oven).

### Karl Fischer titration

A Karl Fischer automated titrator (Radiometer Copenhagen TIM 900) with ethanol based HYDRANAL reagents was used to measure moisture content of ILs after drying and prior to use.

### Preliminary dissolution experiments

Non-extracted bagasse was treated in [C4mim]Cl, [C2mim]Cl, [C2mim]OAc under conditions used in previously reported work [10] (150°C, 90 min and 5% mass bagasse in IL). The treated mixture (partially dissolved bagasse in IL) was then diluted with DMSO and the undissolved and the dissolved-then-precipitated (with water) solid fractions were recovered, dried and weighed as described in previous work by the authors [10]. The estimates of standard deviation (absolute) for this technique (based on duplicate dissolution experiments, 5 degrees of freedom, (df)) are 6% starting mass for undissolved solids and 3% starting mass for dissolved-then-precipitated solids.

### Mass balance determinations for three IL treatments

Bagasse (0.25 mm – 0.5 mm) was extracted with ethanol and water using a Sohxlet device according to the NREL protocol for biomass extractives [23]. ILs (ca. 30 g of either [C4mim]Cl or [C2mim]Cl or [C2mim]OAc in duplicate) were weighed in sealable pressure glass tubes (ACE glass 50 mL). At this point, IL (ca. 0.5 g) was weighed and set aside for IL recovery analysis (using ion chromatography). Extracted bagasse (3.5% moisture) (ca. 1.5 g for [C4mim]Cl and [C2mim]Cl and 0.75 g for [C2mim]OAc) was added to each pressure tube, sealed with Teflon stoppers and placed in an oil bath which was stabilised at 150°C with magnetic stirring at 200 rpm. Sealing the tubes prevented volatile losses such as acetic acid (b.p. 118.1°C) or furfural (b.p. 161.7°C) from the degradation of xylose. The tubes were left in the oil bath for 60 min (25 min of which at temperature ramp and 35 min at 150°C) and, upon removal, placed in an ice bath with magnetic stirring. After 2 min, the tubes were removed from the ice bath and water was added equal to 0.5 mass fraction of the originally added IL. The tube was sealed again and agitated vigorously until a homogenous solution between water and IL appeared to form. The contents of each tube were quantitatively transferred into a preweighed polypropylene centrifuge tube and centrifuged at 10000 × g for 20 min. The liquid contents of the centrifuge tube were decanted to a new preweighed polypropylene centrifuge tube and weighed (liquid fraction 1 or LF1). The pellet (SF1) was centrifuge washed with distilled water (5 × 30 mL at 10000 × g and 5 min - 10 min cycles), freeze dried overnight (−85°C, 80 mT) and weighed. LF1 1 was precipitated with additional water resulting to a water : IL mass ratio of 2.0. Precipitation and coagulation of solids was aided by storing at 4°C overnight followed by shaker incubating at 55°C - 70°C for 60 min. The resulting precipitate (solid fraction 2 or SF2) was centrifuge washed, freeze dried and weighed. The resulting liquid was acidified to pH ≤1 and mixed with a further 1.5 IL mass equivalents of water to maximise precipitation of lignin in solution. This final precipitate (solid fraction 3 or SF3) was centrifuge washed, freeze dried and weighed.

Losses of liquid components to washings of pellets were accounted for by weighing pellets prior to washing and after drying (it is assumed that the composition of these lost liquid components is the same as the bulk liquid). Similarly, subsampling for analysis was accounted for by careful attention to mass changes.

SF1 and the starting biomass were characterised using the NREL acid hydrolysis protocol [23]. Solid fractions 2 and 3 were characterised for lignin content with the acetyl bromide protocol described by Iiyama and Wallis [24]. The sample of LF1 was directly injected onto the HPLC and the Ion Chromatograph (IC) for quantification of monosaccharides and IL ions respectively while the soluble oligosaccharides were determined by acid hydrolysis. All methods are described in detail in the following sections. The distribution of cellulose, hemicellulose and lignin between solid fractions, liquid fraction monosaccharides and liquid fraction oligosaccharides was finally reported as% mass of the components in the starting material (Table 3). The estimates of standard deviation (absolute, based on duplicate IL pretreatments, 3 df) of the recovery (and analysis) of these components (as%mass starting component) in SF1 are 2% for glucan, 2% for xylan, 3% for arabinan, 1% for acetyl and 2% for lignin (acid soluble + acid insoluble). In LF1, these estimates of standard deviation for the oligosaccharides are 1% for glucan, 2% for xylan, 18% for arabinan and 3% for acetyl and for the monosaccharides they are 0.2% for glucan, 0.2% for xylan, 15% for arabinan and 0.7% for acetyl. The unacceptably high standard deviation for arabinan is attributed to its very low concentrations in the liquid fractions.

### Compositional analysis of “solid fraction 1”

Compositional analysis of SF1 samples was carried out using the standard NREL procedure for determination of structural carbohydrates and lignin in biomass [25]. All samples were freeze dried overnight prior to analysis. Each sample (250 mg) was treated with H_2_SO_4_ (72% mass) at 30°C for 1 h. These samples and a sugar recovery standard (SRS, containing known concentrations of glucose, xylose and arabinose) were then exposed to dilute H_2_SO_4_ (4%) at 121°C for 1 h. The hydrolysis products were determined by HPLC (Waters) equipped with a RI detector (Waters 410) and a Bio-Rad HPX-87 H column operated at 85°C. The mobile phase consisted of 5 mM H_2_SO_4_ with a flow rate of 0.6 mL min^-1^. The glucose, xylose and arabinose results were corrected for acid decomposition using the% mass recovery from the SRS. The polysaccharide and acetyl mass content were calculated by conversion of the monosaccharide and acetic acid results with appropriate multiplication factors (0.90 for glucose, 0.88 for xylose and arabinose, 0.683 for acetic acid). In bagasse, glucan content is considered equal to cellulose content since there is no glucose in the hemicelluloses of sugarcane and sucrose has been removed previously at the sugar mill. The acid- insoluble lignin (AIL) after acid hydrolysis was measured as the mass loss of insoluble residue at 575°C. The acid-soluble lignin (ASL) was measured by UV–vis spectrophotometer (Cintra 40) at 240 nm with an extinction coefficient value of 25 L g^-1^ cm^-1^[25]. Ash was determined by placing separate sample fractions at 575°C.

### Compositional analysis of monosaccharides in liquid fraction 1

Each sample of LF1 (0.5 mL) was weighed in 1.5 mL Eppendorf tubes and diluted with water (0.5 mL). The contents were vortexed thoroughly, filtered through a 0.45 μm nylon filter and injected to a Waters HPLC as described earlier. Glucose, xylose, arabinose and acetic acid masses were converted to glucan, xylan, arabinan and acetate masses using appropriate multiplication factors (as listed earlier). In addition, it was assumed that the detected hydroxymethylfurfural (HMF) and furfural were products of cellulose and xylan degradation respectively. Therefore, HMF and furfural masses were converted to cellulose and xylan mass equivalents using multiplication factors of 1.28 and 1.38 respectively [23].

### Compositional analysis of oligosaccharides in liquid fraction 1

Each sample of LF1 (0.5 mL) and SRS solution (0.5 mL) were weighed in 2 mL twist-top Eppendorf tubes, diluted with water (1 mL) and acidified (with 72% mass H_2_SO_4_) to a pH of 0.3. The contents were vortexed thoroughly and autoclaved (121°C for 60 min; autoclaving did not affect mass). After cooling to room temperature, the autoclaved tube contents were filtered through a 0.45 μm nylon filter and injected onto the HPLC system described earlier. After SRS correction for acid decomposition of sugars and subtraction of the monosaccharide composition results, the difference was converted to polysaccharide mass equivalents (using appropriate multiplication factors as listed earlier) in order to arrive at the composition of the soluble oligosaccharides in LF1.

### ATR-FTIR

A small amount of freeze dried fibre, enough to cover the surface of the probe, was placed on the diamond probe of a Thermo Nicolet 870 FTIR (software: OMNIC 7.3). The sample was pressed with an anvil to increase the surface contacting the probe. Sixty-four scans were acquired for each spectrum and the two replicate spectra for each sample were overlayed. No differences in the replicate spectra of this study were observed and thus only the first spectrum of each sample was used for analysis.

### Acetyl bromide for lignin quantification in solid fractions 2 and 3

The acetyl bromide method as described by Iiyama and Wallis [24] was used to determine the% mass lignin content of solid fractions 2 and 3. Freeze dried solids (ca. 10 mg) were weighed in glass tubes and acetyl bromide in acetic acid (25% mass, 10 mL) and then perchloric acid (70% mass, 0.1 mL) were added. The tubes were sealed with Teflon screw caps and placed in temperature controlled rotary shaker (70°C and 100 rpm for 30 min). After cooling to room temperature the tubes were opened and 2 M NaOH (10 mL) and then glacial acetic acid (25 mL) were added. After agitation, absorbance (280 nm, quartz cuvettes, Cintra UV spectrometer) was measured against glacial acetic acid. The resulting solution was analysed with a Cintra-40 UV spectrometer and the absorbance was referenced to a cuvette with glacial acetic acid. Dilutions with glacial acetic acid were necessary for some samples so that the absorbance was <1.0. The absorbance was converted to percent mass concentration of lignin using an extinction coefficient of 25 L·g^-1^·cm^-1^. The extinction coefficient was determined with the use of a calibration curve based on bagasse of known lignin content. The estimate of standard deviation (absolute) of this technique (duplicate samples of untreated bagasse and soda lignin, 4 df) (as% dry mass of solid analysed) is 3.

### Enzymatic saccharification of solids from 3 IL treatments

Enzymatic hydrolysis experiments (SF1 fraction) were performed in 20 mL scintillation vials on a rotary shaker (150 rpm, 50°C) in volumes of 5 mL with a biomass load of 50 mg cellulose equivalent and Accelerase 1000 (Genencor) activity of 15 FPU g^-1^ (25 μL of Accelerase as received) in 50 mM citrate buffer (pH 4.7). Samples (0.2 mL) were periodically removed, placed in ice, then in boiling water (2 min) and centrifuged. Cellobiose, glucose and xylose concentrations were measured by HPLC (HPLC system as described earlier except a Shodex SPO-810 HPLC column at 85°C with a mobile phase of ultrapure water at 0.6 L min^-1^ were used.). The glucose and cellobiose results were converted to glucan mass equivalents and xylose was converted to xylan mass equivalents using appropriate multiplication factors.

The estimates of standard deviation (absolute) of this analysis (based on duplicate IL pretreatments’ saccharification extents at different time points, 18 df) are 2% mass of glucan and 0.9% mass of xylan.

### Recovery of IL

IL set aside at the start of mass balance experiments (starting IL) was brought to a volume of 50 mL with deionised water. Similarly, known masses of LF1 were diluted with deionised water and injected onto the ion chromatograph (Metrohm 761 with a conductivity detector). For cation analysis, samples were injected onto a Metrosep C 2 150 (150 mm × 4 mm) column with an aqueous mobile phase (25% volume acetone, 6 mM tartaric acid and 0.75 mM dipicolinic acid) at 1 mL min^-1^. For anion analysis samples were injected onto a Metrosep ASupp5 (150 mm × 4 mm) column with an aqueous mobile phase (1 mM NaHCO_3_ and 3.2 mM Na_2_CO_3_) at 0.7 mL min^-1^ and suppressed by post-column addition of H_2_SO_4_ (50 mM). IL mass balance was determined from the results of these analyses. The estimate of standard deviation (absolute) of this technique (as% mass of ions in starting IL) for both cations and anions and for all 3 ILs is 2 (based on duplicate IL pretreatments, 6 df).

## Abbreviations

IL: Ionic Liquid; [C4mim]Cl: 1-butyl-3-methylimidazolium chloride; [C2mim]Cl: 1-ethyl-3-methylimidazolium chloride; [C2mim]OAc: 1-ethyl-3-methylimidazolium acetate.

## Competing interests

The authors declare that they have no competing interests.

## Authors’ contributions

All work has been carried out by SK under the supervision of LE and WD. All authors read and approved the final manuscript.
